# Enzymatic approach to cascade synthesis of bis(indolyl)methanes in pure water†

**DOI:** 10.1039/c9ra10014h

**Published:** 2020-03-16

**Authors:** Yajie Fu, Zeping Lu, Ke Fang, Xinyi He, Huajin Xu, Yi Hu

**Affiliations:** State Key Laboratory of Materials-Oriented Chemical Engineering, School of Pharmaceutical Sciences, Nanjing Tech University Nanjing 210009 China huyi@njtech.edu.cn dg1224097@smail.nju.edu.cn

## Abstract

A mild, efficient, and green protocol was developed for the synthesis of bis(indolyl)methanes catalyzed by lipase TLIM through the cascade reactions of indole with aldehydes in pure water. This methodology offers many superiorities such as excellent yields, wide substrate range, simple procedure, reusable and minimal amount of catalyst, and the ability to be scaled up.

## Abstract 2

TLIM: lipase from *Thermomyces lanuginosus* immobilized on particle silica gel.

## Graphical abstract

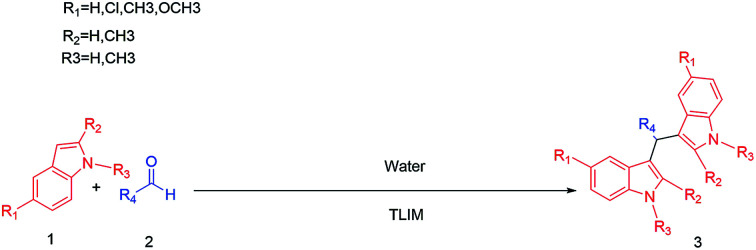

## 1. Introduction

Bis(indolyl)methanes make great contributions in the fields of pharmacological industry and material science. They may act as agonists of the immunostimulatory orphan G protein-coupled receptor GPR84,^1^ as antagonists of *Leishmania donovani* promastigotes as well as axenic amastigotes,^2^ and also as probes for monitoring the interactions of related molecules with transport and response-mediating proteins.^3^ In addition, bis(indolyl)methanes play a brilliant role in the treatment of prostate cancer,^4^ colon cancer,^5^ pancreatic cancer^6^ and breast cancer.^7^ Bis(indolyl)methanes are generally obtained from cascade reactions of indole with carbonyl compounds catalyzed by protic or Lewis acids, which may be easily captured by nitrogenous compounds so as to bring some problems for the synthesis of bis(indolyl)methanes.^8^ Recently, various novel catalysts have been reported to synthesize bis(indolyl)methanes, such as aza-crown ether ionic liquids supported on magnetic Fe_3_O_4_@SiO_2_ core–shell particles,^9^ nanocomposites ZrO_2_–Al_2_O_3_–Fe_3_O_4_,^10^ 1-hexenesulphonic acid sodium salt under ultrasound irradiation,^11^ SDS,^12^ zeolite,^13^ graphene oxide,^14^ AgOTf,^15^ LASSC catalytic system,^16^ meglumine catalyst,^17^ NADES,^18^*etc.* However, there are some shortcomings in the most of catalytic methods mentioned above including expensive or complicated catalyst used, relative high temperature and excessive substrate needed, volatile organic solvent employed, *etc.* Therefore, it is meaningful to develop a green and efficient method to synthesize bis(indolyl)methanes.

The application of lipase in C–C bond formation reaction is attracting more and more attention due to its mild reaction conditions, lack of coenzyme requirements and the ability to work not only in aqueous systems but also in organic solvents.^19^ PPL was employed in aqueous 1,4-dioxane to catalyze the cascade reactions of indole with aromatic aldehydes to synthesize bis(indolyl)methanes.^20^ Le *et al.*^21^ found that α-chymotrypsin in aqueous ethanol exhibited prominent promiscuity for the synthesis of bis(indolyl)methanes. However, both of them could only obtain low to moderate yields for reactions of indole with aromatic aldehydes bearing electron-donating substituents as well as aliphatic aldehydes, and large amount of non-recyclable lipase was needed. In continuation of our interest in lipase-catalyzed C–C bond forming reactions,^22^ we herein report that cascade reactions of indole with aromatic aldehydes can be catalyzed by lipase TLIM in pure water to synthesize bis(indolyl)methanes effectively.

## 2. Materials and methods

### 2.1 Materials

Porcine pancreas lipase (PPL), Amano Lipase PS from *Burkholderia cepacia* (BCL) and *Candida rugosa* lipase (CRL) were purchased from Sigma. Lipase from *Thermomyces lanuginosus* immobilized on particle silica gel (TLIM), lipase from *Rhizomucor miehei* immobilized on anion exchange resin (RMIM), lipase B from *Candida antarctica* immobilized on a macroporous acrylic resin (Novozym 435) and papain were purchased from Novo Nordisk. Bovine serum albumin (BSA) was purchased from Shanghai Huixing. Other reagents were commercially available and were used without further purification.

### 2.2 Characterization

The melting points were determined on a WRS-1B digital melting point instrument and were not corrected. The ^1^H NMR and ^13^C NMR spectra were measured on a Bruker Advance 2B 300 MHz instrument with CDCl_3_ as solvent and TMS as internal standard. The HRMS were measured on Agilent LC/MS mass spectrometer. The progress of the reaction was monitored by TLC using pre-coated Haiyang GF254 silica gel plates. HPLC data was obtained using Dionex Liquid Chromatography (Diamonsil C18(2) (4.6 × 250 mm, 5 μm), water with 0.1% formic acid/methanol (v/v = 1/4, 20 min, radiant elution), 40 °C, 254 nm).

### 2.3 General procedure for the synthesis of bis(indolyl)methanes

A mixture of indole (2 mmol), aldehyde (1 mmol) and lipase (10 mg) in pure water (5 mL) was stirred at 55 °C. The progress of the reaction was monitored by TLC (ethyl acetate–petroleum ether, 1/2). Upon completion of the reaction, the reaction mixture was filtered, and the residue achieved was then dissolved in 1,4-dioxane (10 mL) to separate the product and lipase. By simple filtration, lipase was recovered and applied to the next run directly. After evaporation, to recover 1,4-dioxane, the crude products could be obtained and further purified by column chromatography (eluent, ethyl acetate–petroleum ether, 1/3) on silica gel (200–300 mesh).

## 3. Results and discussion

### 3.1 Screening of enzyme sources for the cascade synthesis of bis(indolyl)methanes

**Scheme 1:**
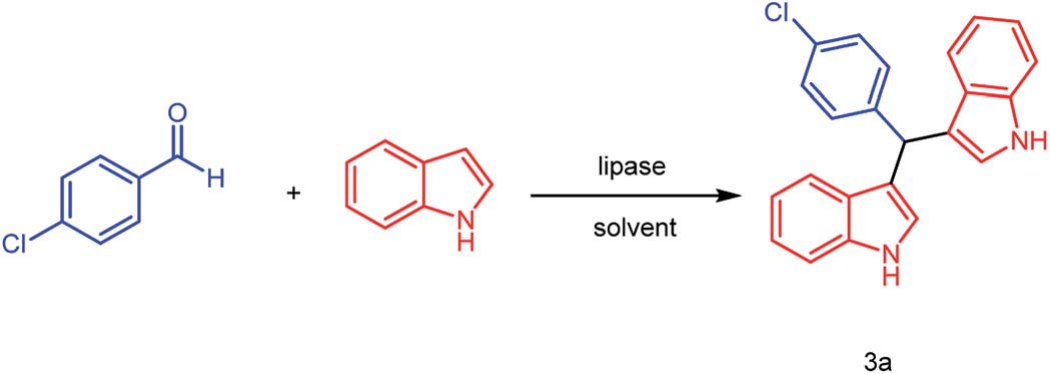
Enzymatic reaction of *p*-chlorobenzaldehyde with 1*H*-indole.

In our previous research,^22*d*^ lipase RMIM was successfully used in pure water to catalyze the Knoevenagel–Michael cascade reactions of 4-hydroxycoumarin with aromatic, heterocyclic or aliphatic aldehydes to synthesize dicoumarol derivatives (81–98%). In our initial research, the cascade reaction of 1*H*-indole with 4-chlorobenzaldehyde was catalyzed by lipase RMIM in pure water (Table 1, entry 1). However, the result was not as satisfactory as we expected and only 51% of product was obtained. Subsequently, we tried other lipases to screen more effective catalysts. As showed in Table 1, lipases CRL, BCL, Novozym 435, PPL, TLIM (Table 1, entries 3–7) as well as papain (Table 1, entry 9) and BSA (Table 1, entry 10) can promote the model reaction (Scheme 1). Lipase TLIM (lipase from *Thermomyces lanuginosus*, immobilized on particle silica gel, the catalyst dosage of lipase on particle silica gel is 7%) showed the best catalytic activity and gave the corresponding product in 56% yield. As for the denatured TLIM (Table 1, entry 8), it demonstrated little catalytic activity and presented similar result with the blank control reaction (Table 1, entry 2). We supposed that the specific tertiary structure of enzymes makes great contributions in this enzyme-catalyzed reaction. The immobilized lipase TLIM is relatively efficient and has the possibility of reuse since it worked as a heterogeneous catalyst. As a result, lipase TLIM was chosen as the promoter (Table 2).

**Table 1:** Effect of enzyme sources on the yield of 3a

Entry	Enzyme^a^	Yield^b^ (%)	Entry	Enzyme^a^	Yield^b^ (%)
1	RMIM	51	6	PPL	49
2	No enzyme	3	7	TLIM	56
3	CRL	37	8	TLIM^c^	7
4	BCL	40	9	Papain	40
5	Novozym 435	43	10	BSA	41

a Reaction conditions: lipase (50 mg), 4-chlorobenzaldehyde (1 mmol), 1*H*-indole (2 mmol), water (5 mL), 45 °C, 18 h.

b HPLC yield.

c Denatured TLIM was obtained by treating with acetone for 24 h.

**Table 2:** Effect of solvent source on the yield of 3a

Entry	Solvent^a^	Yield^b^ (%)
1	*n*-Hexane	12
2	Toluene	51
3	Water	56
4	2-Propanol	0
5	Acetonitrile	0
6	1,4-Dioxane	0
7	DMF	0
8	THF	0
9	DMSO	0
10	Ethanol	Trace

a Reaction conditions: TLIM (50 mg), 4-chlorobenzaldehyde (1 mmol), 1*H*-indole (2 mmol), solvent (5 mL), 45 °C, 18 h.

b HPLC yield.

### 3.2 Screening of solvent for the cascade synthesis of bis(indolyl)methanes

In our previous research,^22*c*^ lipase TLIM showed the best result in *n*-hexane to catalyze the Knoevenagel–Michael cascade reactions of 1,3-diketones with aromatic aldehydes to generate 80–97% yields of xanthone derivatives. While in this study, TLIM showed better catalytic activity in water, and only 12% yield could be obtained *n*-hexane. Additionally, comparable yield also could be obtained when toluene was used as reaction media. Surprisingly, no product could be detected in solvents having a good solubility for the substrates and products like 2-propanol, acetonitrile, DMF, THF and DMSO. PPL was employed in aqueous 1,4-dioxane to synthesize bis(indolyl)methanes.^20^ Le *et al.*^21^ found that α-chymotrypsin in aqueous ethanol exhibited prominent promiscuity for the synthesis of bis(indolyl)methanes. However, no product in 1,4-dioxane and trace product in ethanol were detected in this research. Water has the worst solubility for the substrates and products, which may contribute to the progress of the reaction.

### 3.3 Screening of temperature, enzyme amount and reaction time for the cascade synthesis of bis(indolyl)methanes

**Fig. 1:**
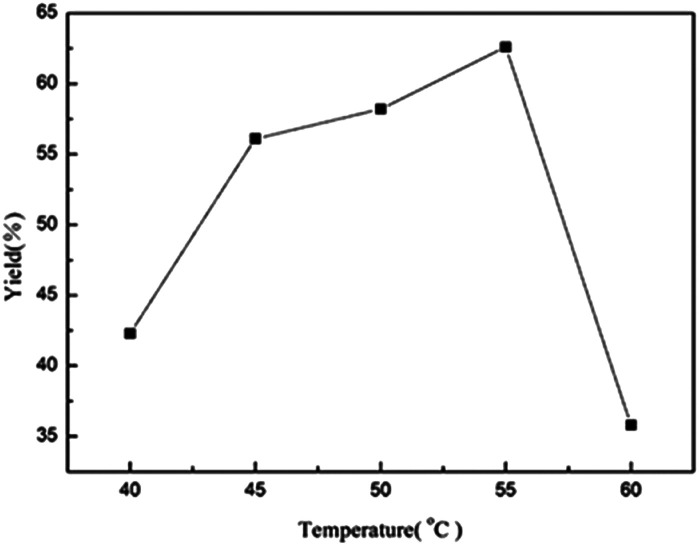
Effects of temperature on the yield of 3a. Reaction conditions: TLIM (50 mg), 4-chlorobenzaldehyde (1 mmol), 1*H*-indole (2 mmol), water (5 mL), 18 h, HPLC yield.

The activity of enzymes and the rate of reactions are strongly associated with the temperature of reactions. The most suitable reaction temperature for TLIM in pure water to produce bis(indolyl)methanes is 55 °C (Fig. 1). Subsequently, we investigated the effect of enzyme loading on this cascade reaction. The results seem to be equivalent when the enzyme amount is more than 10 mg (Table 3, entries 2–4). As a result, 10 mg was chosen to be the optimized enzyme loading for 1 mmol 4-chlorobenzaldehyde substrate of this reaction, which is noteworthy that the enzyme amount is much lesser than other enzymatic reactions.^20,21^ With the optimized conditions in hand, high yields (93%) of 3a could be obtained after 36 h (Table 3, entry 7).

**Table 3:** Effects of enzyme amount and reaction time on the yield of 3a

Entry^a^	Enzyme amount (mg)	Time (h)	Yield^b^ (%)
1	5	18	57
2	10	18	73
3	20	18	68
4	30	18	70
5	10	24	82
6	10	30	88
7	10	36	93
8	10	48	92

a Reaction conditions: TLIM, 4-chlorobenzaldehyde (1 mmol), 1*H*-indole (2 mmol), water (5 mL), 55 °C.

b HPLC yield.

### 3.4 TLIM-catalyzed synthesis of bis(indolyl)methanes

**Scheme 2:**
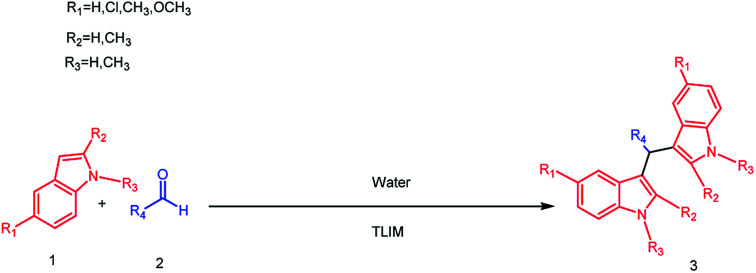
Enzymatic cascade reaction of aldehydes with indole.

Considering the overall effects of catalyst, solvents, temperature and catalyst loading were investigated, further studies on the exploration of substrate scopes and limitations were carried out (Scheme 2). As can be seen in Table 4, aromatic aldehydes with electron-withdrawing substituents (Table 4, entries 1–5) as well as aromatic aldehydes with electron-donating substituents (Table 4, entries 6–12) can both react easily with 1*H*-indole to give corresponding products with 75–99% yields. It is worth noting that the reported enzymatic cascade reactions couldn't get satisfactory yields when aliphatic aldehydes and aromatic aldehydes with electron-donating substituents react with 1*H*-indole.^20,21^ It is extraordinary that excellent result was obtained for aromatic aldehydes having large steric hindrance such as 4-*tert*-butylbenzaldehyde (Table 4, entry 15) with 1*H*-indole after 72 h. Satisfactorily, heterocyclic aldehydes which haven't been investigated in the previous enzymatic synthesis^20,21^ like 2-thiophenealdehyde (Table 4, entry 13) and pyridine-2-carbaldehyde (Table 4, entry 14) could react easily with 1*H*-indole to generate corresponding products in good yields after 72 h. In addition, indole bearing different substituents (Table 4, entries 16–20) also could react smoothly with aldehydes under the reaction conditions with excellent yields obtained. Practically, this protocol could be applied to a gram-scale synthesis and high yield was obtained (Table 4, entry 21). Finally, recyclability and reusability of TLIM were investigated for the synthesis of 3a in a good yields (Table 4, entries 22–23). In summary, the method developed in this paper has a wider substrate scopes than the reported enzymatic cascade reactions and lipase TLIM could be reused with little loss of activity.

**Table 4:** TLIM-catalyzed synthesis of bis(indolyl)methanes

Entry^a^	R_1_	R_2_	R_3_	R_4_	Product	Time/h	Yield^b^/%
1	H	H	H	4-ClC_6_H_5_	3a	36	93(90^d^)
2	H	H	H	3-NO_2_-C_6_H_5_	3b	36	85
3	H	H	H	4-NO_2_-C_6_H_5_	3c	36	87
4	H	H	H	2-F-C_6_H_5_	3d	36	99
5	H	H	H	4-CN-C_6_H_5_	3e	36	98
6	H	H	H	C_6_H_6_	3f	36	98
7	H	H	H	4-CH_3_-C_6_H_5_	3g	36	95
8	H	H	H	4-OCH_3_-C_6_H_5_	3h	36	95
9	H	H	H	4-OH-3-OCH_3_-C_6_H_4_	3i	36	88
10	H	H	H	2-OH-3-OCH_3_-C_6_H_4_	3j	36	88
11	H	H	H	C_3_H_7_	3k	72	87
12	H	H	H	H^c^	3l	36	75
13	H	H	H	2-Thienyl	3m	72	89
14	H	H	H	2-Pyridyl	3n	72	72
15	H	H	H	4-*t*-C_4_H_9_-C_6_H_5_	3o	72	95
16	H	CH_3_	H	4-Cl-C_6_H_5_	3p	36	96^d^
17	CH_3_	H	H	4-Cl-C_6_H_5_	3q	36	98^d^
18	OCH_3_	H	H	4-Cl-C_6_H_5_	3r	36	88^d^
19	Cl	H	H	4-Cl-C_6_H_5_	3s	36	90^d^
20	H	H	CH_3_	4-Cl-C_6_H_5_	3t	36	93^d^
21	H	H	H	4-Cl-C_6_H_5_	3a	36	94^e^
22	H	H	H	4-Cl-C_6_H_5_	3a	36	86^f^
23	H	H	H	4-Cl-C_6_H_5_	3a	36	81^g^
24	H	H	H	4-Cl-C_6_H_5_	3a	36	77^h^
25	H	H	H	4-Cl-C_6_H_5_	3a	36	74^i^

a Reaction conditions: TLIM (10 mg), aldehyde (1 mmol), indole (2 mmol), water (5 mL), 55 °C.

b HPLC yield.

c Reaction conditions: TLIM (10 mg), formaldehyde aqueous solution (37%, 3 mmol), indole (2 mmol), water (5 mL), 55 °C.

d Isolated yield.

e Reaction conditions: TLIM (100 mg), 4-chlorobenzaldehyde (10 mmol), 1*H*-indole (20 mmol), water (50 mL), 55 °C, HPLC yield.

f HPLC yield of 3a (run 2).

g HPLC yield of 3a (run 3).

h HPLC yield of 3a (run 4).

i HPLC yield of 3a (run 5).

### 3.5 Possible mechanism for the synthesis of bis(indolyl)methanes catalyzed by TLIM

**Scheme 3:**
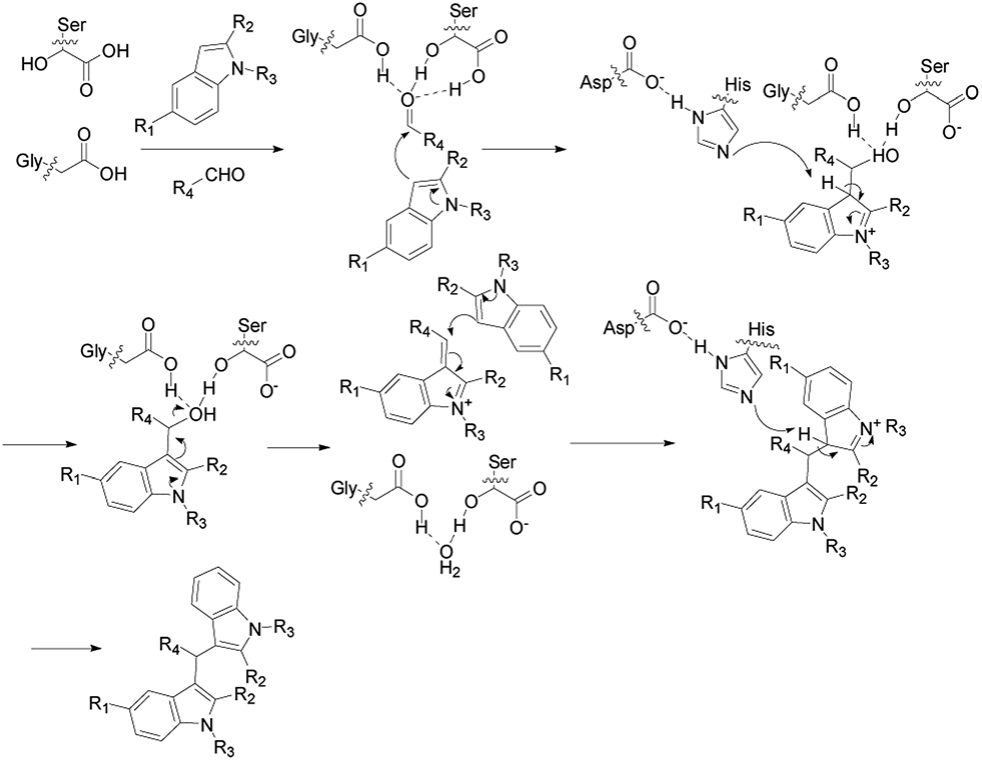
Possible mechanism for the synthesis of bis(indolyl)methanes catalyzed by TLIM.

Enzymatic synthesis of bis(indolyl)methanes was carried out using lipase TLIM as catalyst. Based on literatures,^18,20^ it can be explained by a proposed mechanism depicted in Scheme 3. We supposed that the catalytic active site of lipase TLIM plays a vital role in the cascade reaction. Firstly, the Gly residues and Ser residues interacted with the carbonyl of aromatic aldehyde to form the oxyanion hole, which could stabilized the activated aromatic aldehyde. Then the highly nucleophilic 3-position of indole attacked the activated aromatic aldehyde to form the intermediate product, which interacted with His residues. Finally, the intermediate product contacted with another molecule of indole and joined together to form the final product and released a molecule of water, and the catalyst was set free for the next cycle.

## 4. Comparison of the catalytic efficiency of TLIM with some reported catalysts

Finally, we selected the reaction of 4-methoxybenzaldehyde with indole for the synthesis of 3h as a model reaction and compared the catalytic efficiency of TLIM with other reported catalysts in terms of reaction time, temperature, solvent and yields (Table 5). As can be seen in Table 5, this work could generate higher yield of corresponding product than most of reported methods. Moreover, TLIM was used in pure water instead of volatile, toxic organic solvents. Compared with chemical catalysis methods, TLIM-catalyzed synthesis of bis(3-indolyl)methane has several advantages such as environmental begin, simple, commercially-available and recyclable catalyst.

**Table 5:** Comparison of the catalytic efficiency of TLIM with some reported catalysts

Entry	Catalyst	Time/h	Temperature/°C	Solvent	Yield/%	Ref.
1	Lipase TLIM	36	55	Water	95	This work
2	SDS	18	rt	MeOH	78	12
3	Zeolites	1.5	rt	CH_2_Cl_2_	85	13
4	Lipase PPL	72	50	Aqueous 1,4-dioxane	50	20
5	α-Chymotrypsin	32	50	Aqueous ethanol	75	21
6	MoS_2_–RGO	2	rt	Water	89	23
7	[DABCO-H][HSO_4_]	2	90	—	74	24

## 5. Spectroscopic data for new products

### 5.1 3,3′-((2-Fluorophenyl)methylene)bis(1*H*-indole) (3d)

Yield: 0.99 mmol (99%); red solid; mp 76–77 °C. ^1^H NMR (300 MHz, CDCl_3_) *δ* 7.93 (s, 2H), 7.38 (dd, *J* = 13.0, 8.1 Hz, 4H), 7.24–7.13 (m, 4H), 7.09 (d, *J* = 9.6 Hz, 1H), 7.00 (q, *J* = 6.9, 6.1 Hz, 3H), 6.73 (s, 2H), 6.23 (s, 1H). ^13^C NMR (75 MHz, CDCl_3_) *δ* 160.2, 158.2, 137.5, 129.8, 129.6, 128.6, 127.4, 125.6, 122.7, 121.2, 119.2, 115.4, 114.3, 111.6, 30.8. HRMS (EI-TOF): *m*/*z* calcd for C_23_H_17_N_2_F [M + Na]^+^: 340.3921, found 340.3930.

### 5.2 3,3′-((4-Chlorophenyl)methylene)bis(2-methyl-1*H*-indole) (3p)

Yield: 0.3686 g (96%); red solid; mp 162–163 °C. ^1^H NMR (300 MHz, CDCl_3_) *δ* 7.78 (s, 2H), 7.28 (s, 2H), 7.24 (d, *J* = 8.9 Hz, 4H), 7.07 (t, *J* = 7.4 Hz, 2H), 7.00 (d, *J* = 7.8 Hz, 2H), 6.94–6.86 (m, 2H), 5.98 (s, 1H), 2.07 (s, 6H). ^13^C NMR (75 MHz, CDCl_3_) *δ* 135.0, 131.8, 130.4, 129.3, 128.2, 120.7, 119.2, 112.9, 110.0, 104.6, 38.7, 12.4. HRMS (EI-TOF): *m*/*z* calcd for C_25_H_21_N_2_Cl[M + Na]^+^: 384.1400, found 384.1389.

### 5.3 3,3′-((4-Chlorophenyl)methylene)bis(5-chloro-1*H*-indole) (3s)

Yield: 0.3816 g (90%); pink solid; mp 175–176 °C. ^1^H NMR (300 MHz, CDCl_3_) *δ* 7.96 (s, 2H), 7.30 (d, *J* = 7.2 Hz, 4H), 7.26 (s, 2H), 7.22 (d, *J* = 8.5 Hz, 2H), 7.19–7.06 (m, 2H), 6.65 (s, 2H), 5.74 (s, 1H). ^13^C NMR (75 MHz, CDCl_3_) *δ* 141.3, 136.8, 128.70, 128.6, 127.4, 127.2, 124.2, 121.2, 120.8, 119.6, 113.7, 112.2, 34.8. HRMS (EI-TOF): *m*/*z* calcd for C_23_H_15_N_2_Cl_3_[M + Na]^+^: 424.0311 found 424.0315.

## 6. Conclusions

In summary, we have successfully developed an environmentally friendly and highly efficient method to synthesize bis(indolyl)methanes, which could make for some drawbacks of the reported enzyme-catalyzed synthesis of bis(indolyl)methanes. This protocol can generate bis(indolyl)methanes with wide substrate range in excellent yields in pure water using very small amount of recyclable lipase. What's more, this enzymatic approach to cascade synthesis of bis(indolyl)methanes could be scaled up.

## Conflicts of interest

There are no conflicts to declare.

## Supplementary Material

RA-010-C9RA10014H-s001
