# Supplementary material for: Enzymatic approach to cascade synthesis of bis(indolyl)methanes in pure water
Source: RSC Adv. 2020 Mar 16;10(18):10848–53. doi: 10.1039/c9ra10014h (PMC9050360; doi:10.1039/c9ra10014h)
Supplement: RA-010-C9RA10014H-s001 [file RA-010-C9RA10014H-s001.pdf]

Electronic Supplementary Material (ESI) for RSC Advances.
This journal is © The Royal Society of Chemistry 2020

Synthesis of Bis(indolyl)methanes; Typical Procedure for 3a:

A mixture of indole (0.2341g, 2mmol), 4-chlorobenzaldehyde (0.1406g, 1mmol) and TLIM (10mg) in pure water (5 mL) was stirred at 55°C. The progress of the reaction was monitored by TLC (ethyl acetate-petroleum ether, 1:2). Upon completion of the reaction, the reaction mixture was filtered, and the residue achieved was then dissolved in 1, 4-dioxane (10mL) to separate the product and TLIM. By simple filtration, the immobilized biocatalyst TLIM was recovered and applied to the next run directly. After evaporation, to recover 1, 4-dioxane, the crude products could be obtained and further purified by column chromatography (eluent, ethyl acetate -petroleum ether, 1/3) on silica gel (200-300mesh).

3,3'-((4-chlorophenyl)methylene)bis(1H-indole)(3a):
Yield: 0.93mmol (93%); red solid; m p 76°C-77°C.
^{1}H NMR (300 MHz, $CDCl_3$) δ 7.92 (s, 2H), 7.35 (dd, *J* = 8.1, 2.7 Hz, 4H), 7.29 – 7.21 (m, 4H), 7.20 – 7.12 (m, 2H), 7.10 – 6.91 (m, 2H), 6.62 (dd, *J* = 2.4, 1.0 Hz, 2H), 5.85 (s, 1H).
^{13}C NMR (75 MHz, $CDCl_3$) δ 138.9 , 131.2 , 129.8 , 128.6 , 127.8 , 127.2 , 121.2 , 121.0 , 119.7 , 119.2 , 112.2 , 111.6 , 34.8 .
HRMS (EI-TOF): m/z Calcd. for $C_{23}H_{17}ClN_2[M+Na]^+$:356.8511, found 356.8514.

3,3'-((3-nitrophenyl)methylene)bis(1H-indole)(3b):
Yield: 0.85mmol (85%); orange solid; m p 86°C-87°C.
^{1}H NMR (300 MHz, $CDCl_3$) δ 10.01 (s, 2H), 8.11 (s, 1H), 7.98 (d, *J* = 8.1 Hz, 1H), 7.88 (s, 1H), 7.59 (d, *J* = 7.7 Hz, 1H), 7.32 (dd, *J* = 14.6, 6.7 Hz, 2H), 7.25 (d, *J* = 6.6 Hz, 2H), 7.09 (t, *J* = 7.5 Hz, 2H), 6.92 (t, *J* = 7.6 Hz, 2H), 6.56 (s, 2H), 5.89 (s, 1H).
^{13}C NMR (75 MHz, $CDCl_3$)δ 148.3 , 137.8 , 133.2 , 129.9 , 127.8 , 127.2 , 125.7 , 121.5 , 121.2 , 121.0 , 119.7 , 119.2 , 112.2 , 111.6 , 34.7 .
HRMS (EI-TOF): m/z Calcd. for $C_{23}H_{17}N_3O_2[M+Na]^+$:367.4009, found 367.3998.

3,3'-((4-nitrophenyl)methylene)bis(1H-indole)(3c):
Yield: 0.87mmol (87%); orange solid; m p 220°C-222°C.
^{1}H NMR (300 MHz, $CDCl_3$) δ 8.14. (d, *J* = 8.6 Hz, 2H), 8.02 (s, 2H), 7.51 (d, *J* = 8.5 Hz, 2H), 7.36 (dd, *J* = 14.8, 8.0 Hz, 4H), 7.20 (t, *J* = 7.5 Hz, 2H), 7.03 (t, *J* = 7.5 Hz, 2H), 6.69 (s, 2H), 5.99 (s, 1H).
HRMS (EI-TOF): m/z Calcd. for $C_{23}H_{17}N_3O_2[M+Na]^+$:367.4011, found 367.3999.

3,3'-((2-fluorophenyl)methylene)bis(1H-indole)(3d):
Yield: 0.99mmol (99%); red solid; m p 76°C-77°C.
^{1}H NMR (300 MHz, $CDCl_3$) δ 7.93 (s, 2H), 7.38 (dd, *J* = 13.0, 8.1 Hz, 4H), 7.24 – 7.13 (m, 4H), 7.09 (d, *J* = 9.6 Hz, 1H), 7.00 (q, *J* = 6.9, 6.1 Hz, 3H), 6.73 (s, 2H), 6.23 (s, 1H).
^{13}C NMR (75 MHz, $CDCl_3$) δ 160.2 , 158.2 , 137.5 , 129.8, 129.6, 128.6, 127.4 , 125.6 , 122.7 , 121.2 , 119.2 , 115.4, 114.3, 111.6 , 30.8 .
HRMS (EI-TOF): m/z Calcd. for $C_{23}H_{17}N_2F$ $[M+Na]^+$:340.3921, found 340.3930.

4-(di(1H-indol-3-yl)methyl)benzonitrile(3e):
Yield: 0.98mmol (98%); red solid; m p 215°C-217°C.
^{1}H NMR (300 MHz, $CDCl_3$) δ 8.00 (s, 2H), 7.57 (d, *J* = 8.2 Hz, 2H), 7.45 (d, *J* = 8.1 Hz, 2H), 7.36 (dd, *J* = 14.4, 8.1 Hz, 4H), 7.20 (t, *J* = 7.6 Hz, 2H), 7.03 (t, *J* = 7.5 Hz, 2H), 6.67 (s, 2H), 5.94 (s, 1H).

^{13}C NMR (75 MHz, $CDCl_3$) δ 149.8 , 136.7 , 132.1 , 129.5 , 126.7 , 123.6 , 122.2 , 119.6 , 119.5 , 119.2 , 118.2 , 111.2 , 110.0 , 40.4 .

HRMS (EI-TOF): m/z Calcd. for $C_{23}H_{17}N_3[M+Na]^+$:335.1430, found 335.1499.

3,3'-(phenylmethylene)bis(1H-indole)(3f):

Yield: 0.98mmol (98%); red solid; m p 124℃-126℃.

^1H NMR (300 MHz, $CDCl_3$) δ 7.88 (s, 2H), 7.48 – 7.33 (m, 6H), 7.30 (d, *J* = 7.0 Hz, 2H), 7.23 – 7.13 (m, 3H), 7.01 (t, *J* = 7.4 Hz, 2H), 6.65 (s, 2H), 5.90 (s, 1H).

HRMS (EI-TOF): m/z Calcd. for $C_{23}H_{18}N_2[M+Na]^+$:322.4013, found 322.4019.

3,3'-(p-tolylmethylene)bis(1H-indole)(3g):

Yield: 0.95mmol (95%); orange solid; m p 97℃-99℃.

^1H NMR (300 MHz, $CDCl_3$) δ 7.66 (s, 2H), 7.42 (d, *J* = 7.8 Hz,2H), 7.26 (t, *J* = 7.3 Hz, 4H), 7.18 (t, *J* = 7.4 Hz, 2H), 7.10 (d, *J* = 7.7 Hz, 2H), 7.03 (t, *J* = 7.3 Hz, 2H), 6.54 (s, 2H), 5.86 (s, 1H), 2.35 (s, 3H).

^{13}C NMR (75 MHz, $CDCl_3$) δ 141.0 , 136.7 , 135.4 , 128.9 , 128.5 , 127.1 , 126.1 , 123.5 , 121.8 , 119.9 , 119.2 , 111.0 , 39.8 , 21.0 .

HRMS (EI-TOF): m/z Calcd. for $C_{24}H_{20}N_2[M+Na]^+$:336.4313, found 336.4311.

3,3'-((4-methoxyphenyl)methylene)bis(1H-indole)(3h):

Yield: 0.95mmol (95%); red solid; m p 187℃-189℃

^1H NMR (300 MHz, $CDCl_3$) δ 7.92 (s, 2H), 7.37 (dd, *J* = 14.3, 8.1 Hz, 4H), 7.25 (d, *J* = 6.3 Hz, 2H), 7.16 (t, *J* = 7.5 Hz, 2H), 7.00 (t, *J* = 7.5 Hz, 2H), 6.82 (d, *J* = 8.5 Hz, 2H), 6.64 (s, 2H), 5.84 (s, 1H), 3.78 (s, 3H).

HRMS (EI-TOF): m/z Calcd. for $C_{24}H_{20}N_2O[M+Na]^+$:352.4322, found 352.4310.

4-(di(1H-indol-3-yl)methyl)-2-methoxyphenol(3i):

Yield: 0.88mmol (88%); red solid; m p 113℃-115℃.

^1H NMR (300 MHz, $CDCl_3$) δ 7.87 (s, 2H), 7.42 (d, *J* = 7.9 Hz, 2H), 7.32 (d, *J* = 8.1 Hz, 2H), 7.18 (t, *J* = 7.5 Hz, 2H), 7.02 (t, *J* = 7.4 Hz, 2H), 6.90 (s, 1H), 6.83 (s, 2H), 6.60 (s, 2H), 5.83 (s, 1H), 3.74 (s, 3H), 2.08 (s, 1H).

HRMS (EI-TOF): m/z Calcd. for $C_{24}H_{20}N_2O_2[M+Na]^+$:368.4335, found 368.4373.

2-(di(1H-indol-3-yl)methyl)-6-methoxyphenol(3j):

Yield: 0.88mmol (88%); red solid; m p 225℃-226℃.

^1H NMR (300 MHz, $CDCl_3$) δ 7.88 (s, 2H), 7.45 (d, *J* = 7.9 Hz, 2H), 7.33 (d, *J* = 8.1 Hz, 2H), 7.15 (t, *J* = 7.5 Hz, 2H), 7.00 (t, *J* = 7.4 Hz, 2H), 6.78 (dd, *J* = 9.9, 2.7 Hz, 1H), 6.74 (d, *J* = 3.1 Hz, 1H), 6.70 (s, 2H), 6.30 (s, 1H), 5.83 (s, 1H), 3.89 (s, 3H), 2.05 (s, 1H).

HRMS (EI-TOF): m/z Calcd. for $C_{24}H_{20}N_2O_2[M+Na]^+$:368.4371, found 368.4373.

3,3'-(butane-1,1-diyl)bis(1H-indole)(3k):

Yield: 0.87mmol (87%); orange solid; m p 153℃-155℃.

^1H NMR (300 MHz, $CDCl_3$) δ 7.57 (s, 2H), 7.46 (d, *J* = 7.8 Hz, 2H), 7.10 (t, *J* = 10.0 Hz, 2H), 7.00 (t, *J* = 7.4 Hz, 2H), 6.89 (p, *J* = 8.9, 8.4 Hz, 2H), 6.81 – 6.56 (m, 2H), 4.34 (t, *J* = 7.4 Hz, 1H), 2.05 (q, *J* = 7.5 Hz, 2H), 1.27 (dt, *J* = 18.0, 9.3 Hz, 2H), 0.82 (t, *J* = 7.3 Hz, 3H).

^{13}C NMR (75 MHz, $CDCl_3$) δ 136.6 , 127.2 , 121.7 , 121.5 , 120.5 , 119.7 , 119.0 , 111.1 , 38.2 , 33.7 , 21.4 , 14.2 .

HRMS (EI-TOF): m/z Calcd. for $C_{20}H_{20}N_2$ $[M+Na]^+$:288.3942, found 288.4051.

3,3'-(ethane-1,1-diyl)bis(1H-indole)（3l）:

Yield: 0.75mmol(75%); white solid; m p 157℃-158℃.

^1H NMR (300 MHz, Chloroform-*d*) δ 7.89 (s, 2H), 7.63 (d, *J* = 7.8 Hz, 2H), 7.36 (d, *J* = 8.1 Hz, 2H), 7.22 – 7.18 (m, 2H), 7.13 – 7.07 (m, 2H), 6.97 – 6.90 (m, 2H), 4.26 (d, *J* = 1.2 Hz, 1H), 0.08 (s, 3H).

HRMS (EI-TOF): m/z Calcd. for $C_{18}H_{16}N_2$ $[M+Na]^+$:260.3315, found 260.3310.

3,3'-(thiophen-2-ylmethylene)bis(1H-indole)(3m):

Yield: 0.89mmol (89%); pink solid; m p150℃-152℃.

^1H NMR (300 MHz, $CDCl_3$) δ 7.90 (s, 2H), 7.47 (d, *J* = 7.9 Hz, 2H), 7.35 (d, *J* = 8.1 Hz, 2H), 7.24 – 7.12 (m, 3H), 7.04 (t, *J* = 7.4 Hz, 2H), 6.92 (d, *J* = 4.5 Hz, 2H), 6.84 (s, 2H), 6.17 (s, 1H).

^{13}C NMR (75 MHz, $CDCl_3$) δ 147.6 , 135.5 , 125.7 , 125.4 , 124.1 , 122.6 , 122.1 , 121.0 , 118.7 , 118.6 , 118.3 , 110.1 , 34.3.

HRMS (EI-TOF): m/z Calcd. for $C_{21}H_{12}N_2S$ $[M+Na]^+$:324.0710, found 324.0796.

3,3'-((1H-pyrrol-2-yl)methylene)bis(1H-indole)(3n):

Yield: 0.72mmol (72%); light yellow solid; m p 208℃-210℃.

^1H NMR (300 MHz, $CDCl_3$) δ 8.59 (d, *J* = 4.2 Hz, 1H), 8.06 (s, 2H), 7.60 (t, *J* = 8.3 Hz, 1H), 7.37 (dd, *J* = 15.3, 8.0 Hz, 5H), 7.15 (q, *J* = 5.5, 3.8 Hz, 3H), 7.00 (t, *J* = 7.4 Hz, 2H), 6.82 – 6.77 (m, 2H), 6.08 (s, 1H).

HRMS (EI-TOF): m/z Calcd. for $C_{22}H_{17}N_2$$[M+Na]^+$:309.1412, found 309.1417.

3,3'-((4-(tert-butyl)phenyl)methylene)bis(1H-indole)(3o):

Yield: 0.95mmol (95%); red solid; m p 70℃-72℃.

^1H NMR (300 MHz, $CDCl_3$) δ 7.59 (s, 2H), 7.39 (d, *J* = 7.8 Hz, 2H), 7.35 – 7.18 (m, 6H), 7.13 (t, *J* = 7.4 Hz, 2H), 6.98 (t, *J* = 7.4 Hz, 2H), 6.50 (s, 2H), 5.83 (s, 1H), 1.30 (s, 9H).

^{13}C NMR (75 MHz, $CDCl_3$) δ 148.7 , 140.8 , 136.7 , 128.2 , 127.2 , 125.0 , 123.5 , 121.8 , 120.0 , 119.1 , 111.0 , 104.5 , 39.6 , 34.4 , 31.4 .

HRMS (EI-TOF): m/z Calcd. for $C_{27}H_{26}N_2$$[M+Na]^+$:378.5124, found 378.5087.

3,3'-((4-chlorophenyl)methylene)bis(2-methyl-1H-indole)(3p):

Yield: 0.3686g（96%）; red solid; m p 162℃-163℃.

^1H NMR (300 MHz, $CDCl_3$) δ 7.78 (s, 2H), 7.28 (s, 2H), 7.24 (d, *J* = 8.9 Hz, 4H), 7.07 (t, *J* = 7.4 Hz, 2H), 7.00 (d, *J* = 7.8 Hz, 2H), 6.94 – 6.86 (m, 2H), 5.98 (s, 1H), 2.07 (s, 6H).

^{13}C NMR (75 MHz, $CDCl_3$) δ 135.0 , 131.8 , 130.4 , 129.3 , 128.2 , 120.7 , 119.2 , 112.9 , 110.0 , 104.6 , 38.7 , 12.4 .

HRMS (EI-TOF): m/z Calcd. for $C_{25}H_{21}N_2Cl$$[M+Na]^+$:384.1400, found 384.1389.

3,3'-((4-chlorophenyl)methylene)bis(5-methyl-1H-indole)(3q):

Yield:0.3763g (98%); red solid; m p 148℃-150℃.

^1H NMR (300 MHz, $CDCl_3$) δ 7.76 (s, 2H), 7.24 (d, *J* = 9.2 Hz, 6H), 7.18 (s, 2H), 7.03 (d, *J* = 8.2 Hz,

2H), 6.56 (s, 2H), 5.81 (s, 1H), 2.38 (s, 6H).

^{13}C NMR (75 MHz, $CDCl_3$) δ 143.2, 134.2, 131.7, 131.3, 128.8, 128.4, 127.3, 124.0, 122.9, 119.3, 119.0, 110.6, 40.3, 21.2

HRMS (EI-TOF): m/z Calcd. for $C_{25}H_{21}N_2Cl[M+Na]^+$:384.1400, found 384.1389.

3,3'-((4-chlorophenyl)methylene)bis(5-methoxy-1H-indole)(3r):

Yield: 0.3661g (88%); pink solid; m p 155℃-157℃.

^{1}H NMR (300 MHz, $CDCl_3$) δ 7.84 (s, 2H), 7.27 (d, *J* = 5.1 Hz, 4H), 7.23 (s, 2H), 6.95 – 6.76 (m, 4H), 6.63 (s, 2H), 5.77 (s, 1H), 3.73 (s, 6H).

^{13}C NMR (75 MHz, $CDCl_3$) δ 153.8 , 142.5, 134.7 , 130.1 , 128.3 , 127.3 , 124.4 , 118.7 , 112.0 , 111.8 , 110.9 , 102.0 , 55.9 , 39.7 .

HRMS (EI-TOF): m/z Calcd. for $C_{25}H_{21}N_2O_2Cl[M+Na]^+$:416.1299 found 416.1301.

3,3'-((4-chlorophenyl)methylene)bis(5-chloro-1H-indole)(3s):

Yield:0.3816g (90%); pink solid; m p 175℃-176℃.

^{1}H NMR (300 MHz, $CDCl_3$) δ 7.96 (s, 2H), 7.30 (d, *J* = 7.2 Hz, 4H), 7.26 (s, 2H), 7.22 (d, *J* = 8.5 Hz, 2H), 7.19 – 7.06 (m, 2H), 6.65 (s, 2H), 5.74 (s, 1H).

^{13}C NMR (75 MHz, $CDCl_3$) δ 141.3 , 136.8 , 128.7 , 128.6 , 127.4 , 127.2 , 124.2 , 121.2 , 120.8 , 119.6 , 113.7 , 112.2 , 34.8 .

HRMS (EI-TOF): m/z Calcd. for $C_{23}H_{15}N_2Cl_3[M+Na]^+$:424.0311, found 424.0315.

3,3'-((4-chlorophenyl)methylene)bis(1-methyl-1H-indole)(3t):

Yield: 0.3571g (93%); white solid; m p 208℃-209℃.

^{1}H NMR (400 MHz, DMSO-d_6) δ 7.39 – 7.27 (m, 8H), 7.14 – 7.08 (m, 2H), 6.94 – 6.89 (m, 2H), 6.82 (s, 2H), 5.86 (s, 1H), 3.69 (s, 6H).

HRMS (EI-TOF): m/z Calcd. for $C_{25}H_{21}N_2Cl[M+Na]^+$:384.1422 found 384.1428.
